# Systematic Review of Rehabilitation in Focal Dystonias: Classification and Recommendations

**DOI:** 10.1002/mdc3.12574

**Published:** 2018-03-13

**Authors:** Cecília N. Prudente, Lena Zetterberg, Annika Bring, Lynley Bradnam, Teresa J. Kimberley

**Affiliations:** ^1^ Division of Physical Therapy Department of Rehabilitation Medicine University of Minnesota Minneapolis MN USA; ^2^ Department of Neuroscience Section of Physiotherapy Uppsala University and University Hospital Uppsala Sweden; ^3^ Graduate School of Health University of Technology Sydney Sydney Australia; ^4^ Department of Physical Therapy MGH Institute of Health Professions Boston MA USA

**Keywords:** focal dystonia, rehabilitation, systematic review

## Abstract

**Background:**

Rehabilitation interventions are rarely utilized as an alternative or adjunct therapy for focal dystonias. Reasons for limited utilization are unknown, but lack of conclusive evidence of effectiveness is likely a crucial factor.

**Methods and Findings:**

The purpose of this systematic review was to determine the level of evidence for rehabilitation interventions in focal dystonias. Rehabilitation interventions were classified based upon the underlying theoretical basis of different approaches, and the strength of evidence for each category was evaluated to identify gaps in the field. Prospective studies using rehabilitation methods in cervical, hand, and foot dystonia were reviewed. The key elements of treatments tested were identified and studies were grouped into six categories based on the theoretical basis of the intervention: (1) movement practice, (2) training with constraint, (3) sensory reorganization, (4) normalization of muscle activity with external techniques, (5) neuromodulation with training, and (6) compensatory strategies. Quality of the body of evidence ranged from very low‐to‐low according to the grades of recommendation, assessment, development, and evaluation (GRADE). Despite inconclusive evidence for these rehabilitation approaches, data suggest that intensive movement practice and neuromodulation combined with motor training should be further explored.

**Conclusions:**

This systematic review presents a novel approach to classify studies of rehabilitation in focal dystonias based on the theoretical basis of intervention. The proposed classification system will move toward a unified theoretical understanding of rehabilitation interventions in dystonia. Moreover, it will help provide recommendations for clinical applications and future investigations.

Dystonia, the third most common movement disorder,^1^ consists of a group of conditions characterized by involuntary patterned movements and abnormal posturing. Dystonia can affect virtually any skeletal muscle and be generalized, segmental, or focal depending on the number of body parts affected. Most patients referred to rehabilitation have focal dystonia affecting the neck, hand, or foot. The prevalence of all focal dystonias combined ranges from 3.8 to 177 per 100,000 persons,^2^ but the rates are likely underestimated due to difficulty with diagnosis. The etiology and pathogenesis of focal dystonias remain unknown, although studies have suggested that the dysfunction may be due to alterations in neuroplasticity, inhibition, and integration within sensorimotor networks.^3^

There is significant disability associated with focal dystonia due to pain and impairment, reduction in participation of activities of daily living (ADLs), and employment problems.^4^, ^5^ Several studies report that the burden of focal dystonia often extends beyond the motor symptoms, with problems such as loss of self‐confidence and independence, depression, social withdrawal, insomnia, and fatigue.^5^, ^6^ The current standard care for focal dystonia consists almost exclusively of botulinum neurotoxin (BoNT) injections, which temporarily inhibit acetylcholine release at the neuromuscular junction of the injected musculature. However, BoNT is not effective for all patients and its effects usually wear off around 8–12 weeks.^5^, ^7^ If BoNT is not an option, either because of lack of response, desire to avoid toxin, or expense, other available interventions include anticholinergic drugs and deep brain stimulation, but not all patients are eligible or can tolerate these treatments.

The impairments of focal dystonias are well within the scope of rehabilitation practice, yet these interventions are rarely utilized as an alternative or adjunct to standard care. Rate of utilization varies widely worldwide. In the United States, few therapists regularly see patients with dystonia in their clinical practice. In a survey in 24 European countries, rehabilitation was easily accessible in only half of the countries surveyed.^8^ In Sweden, where physical therapy is more commonly used in the management of cervical dystonia, it is the second most effective intervention after BoNT according to patients.^9^ People with cervical dystonia have reported physical therapy to be one of the most effective adjunct therapies to standard care, but only 31% of patients have ever received rehabilitation.^5^ The reasons for under‐utilization are unknown, but lack of definitive evidence of effectiveness is likely a crucial factor. Current implementation of rehabilitation treatments in focal dystonia is also hampered by lack of understanding of many therapists regarding the etiology and pathophysiology of the disorder, including awareness that some rehabilitation interventions may worsen the condition.

Advances in the field of dystonia rehabilitation will only occur if efficacy for treatment is demonstrated. Thus, a comprehensive review of available evidence is needed. Prior systematic reviews addressed non‐pharmacological treatments delineated by type of focal dystonia.^10^, ^11^, ^12^ Here we focus instead on rehabilitation approaches independent of dystonia type, with the aim of identifying commonalities in interventions for all focal dystonias. Specifically, the goal of this systematic review was to categorize rehabilitation interventions based upon the common underlying theoretical basis of different approaches, and evaluate the strength of the evidence for each category of intervention. By grouping different studies with similar theoretical basis for the intervention employed, we propose a classification system for rehabilitation approaches in focal dystonia which will help to set future directions for clinical applications and research.

## Methods

### Search Strategy

Using Medline and Web of Science databases, we searched for studies published in English between 01 January 1996 and 01 December 2016. For both databases, the search terms included the following:

1. (dystonia OR focal dystonia OR musician's dystonia OR writer's cramp OR spasmodic torticollis) AND (rehabilitation OR physical therapy OR occupational therapy OR exercise)
2. (dystonia OR focal dystonia OR musician's dystonia OR writer's cramp OR spasmodic torticollis) AND (rehabilitation OR physical therapy OR occupational therapy OR exercise) AND neuromodulation

### Review Process, Selection Criteria, and Methodological Quality

The titles of publications retrieved through the search described above were initially screened for relevance by one of the authors. Then, two reviewers screened the abstracts of selected studies for suitability. Inclusion criteria consisted of: (1) studies of focal dystonia types that are within realm of rehabilitation (i.e., cervical, hand and foot dystonias); (2) use of intervention methods within the expertise of rehabilitation professionals (i.e., non‐pharmacological, non‐surgical) or studies testing neuromodulation combined with motor training; (3) prospective studies. Exclusion criteria consisted of: (1) studies of blepharospasm, laryngeal, oromandibular, axial, segmental or generalized dystonia; (2) studies in which the primary goal was not to test an intervention but rather to test the mechanism of a specific method or disease mechanism; (3) single session interventions; (4) neuromodulation studies without motor training; (5) studies in children; (6) observational and retrospective studies; (7) reviews, editorials, commentaries or expert opinion; (8) conference proceedings and abstracts; or (9) articles with unclear methods or results.

Next, two independent reviewers were assigned each article to determine the study's characteristics and rate its overall quality. When necessary, the authors of a publication were contacted to clarify descriptions of methods or results. Final inclusion of a publication in the systematic review was based on the agreement of at least two reviewers using the inclusion and exclusion criteria.

**Figure 1:**
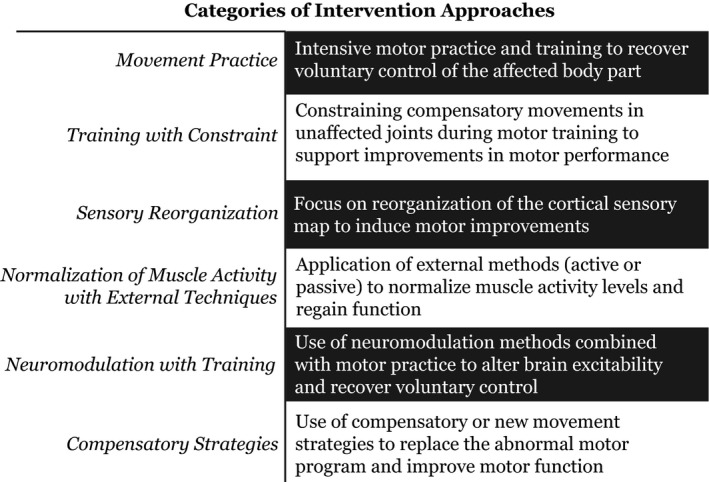
Classification of rehabilitation approaches into categories based on the theoretical basis of interventions.

Following review, the key elements of treatment were identified so that studies with similar intervention approaches could be grouped together. The key factor for grouping studies was the theoretical basis or the primary premise of the intervention tested. It is worth noting that many studies employed a variety of strategies, making classification difficult. In such cases, selection was based on the reviewers’ assessment of the basis for the primary intervention used. Six categories of intervention approaches were identified, based on the key component of the interventions (Figure 1, Supplement 1).

Each category of intervention was rated according to the Grades of Recommendation, Assessment, Development and Evaluation (GRADE) approach which is used to rate the overall quality of evidence and strength of recommendations of studies using similar intervention methods.^13^, ^14^ The quality of evidence indicates the confidence level that an estimate of effect is correct, while the strength of a recommendation indicates how confident we can be that adherence to the recommendation will do more good than harm.^13^ Using the GRADE approach, the overall quality of each category of rehabilitation intervention was classified as high, moderate, low or very low based on the guidelines outlined in Tables 1 and 2. To evaluate each category, the design, quality, consistency, and directness of each study were considered.

**Table 1:** Levels of Body of Evidence Based on the GRADE Approach

Study methods	Quality rating	Description
Randomized trials or double‐upgraded observational studies	High	Further research is unlikely to change confidence in the estimate of effect
Downgraded randomized trials or upgraded observational studies	Moderate	Further research is likely to have important impact in confidence in the estimate of effect and may change the estimate
Observational studies or double‐downgraded randomized trials	Low	Further research is very likely to have important impact in confidence in the estimate of effect and is likely to change the estimate
Case series/case reports, triple‐downgraded randomized trials, or downgraded observational studies	Very low	Any estimate of effect is very uncertain

GRADE: Grades of Recommendation, Assessment, Development and Evaluation. Modified from Atkins, Best^13^ and Guyatt, Oxman^14^

**Table 2:** Criteria for Downgrading or Upgrading Grade of Evidence

Downgrade	Upgrade
Serious (‐1) or very serious (‐2) limitation to study quality Important inconsistency (‐1), i.e., estimate of effects across studies is not consistent Some (‐1) or major (‐2) uncertainty about directness (defined as the extent to which the people, interventions, and outcome measures are similar to those of interest) Imprecise or sparse data (‐1) High probability of reporting bias (‐1)	Strong evidence of association based on consistent evidence from two or more observational studies, with no plausible confounders (+1) Very strong evidence of association based on direct evidence with no major threats of validity (+2) Evidence of a dose‐response gradient (+1) Significant effects or no spurious effects reported despite all plausible confounders (+1) Large magnitude of effect (+1)

Numbers in parentheses represent decrease or increase of quality of evidence level. Modified from Atkins, Best^13^ and Guyatt, Oxman.^14^

## Results

### Overview

**Figure 2:**
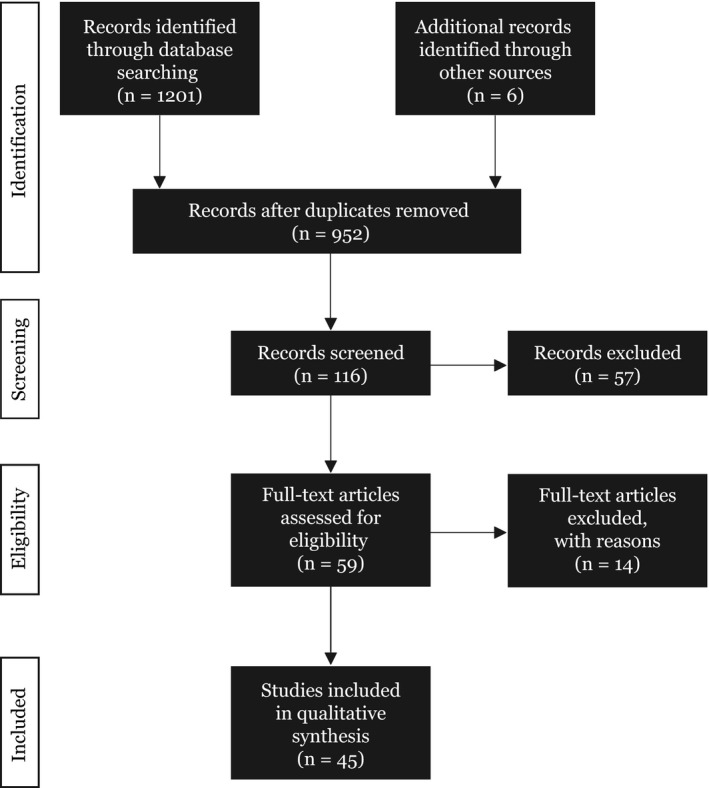
Flow diagram of screening and identification process of the studies reviewed.

The search revealed 1207 publications. Of these, 59 were reviewed by at least two reviewers to determine eligibility and 45 were included in the final review (Figure 2). A variety of study designs were employed in the studies selected, including randomized controlled trials (*n = *8), quasi‐experimental studies (*n = *5), single subject design studies (*n = *4), case series (*n = *21), and case reports (*n = *7). Most studies investigated isolated focal dystonia of adult onset affecting the hand (*n = *36) or neck (*n = *9), while there was only one publication on foot dystonia.

### Intervention Approaches for Rehabilitation of Focal Dystonias

Despite the variety of treatments tested in focal dystonias, common themes emerged from the different interventions reviewed, and six categories of approaches were identified (Supplement 1). Information regarding the studies included in each category is provided below. Of note, statistically significant beneficial effects reported are described as “significant improvements,” while qualitative improvements not tested for statistical significance were described as “improvements.”

#### **Category 1: Movement Practice**

Almost all studies incorporated movement practice as a component of the intervention; however, to be included in this category, the essential element of the treatment must have been intensive motor training, with or without supervision (*n = *12). Movement practice as an intervention for focal dystonias was explored with a variety of methods. Task‐specific motor training was employed most often in writer's cramp^15^ and musician's dystonia^16^, ^17^, ^18^, ^19^ to promote proper body alignment and prevent dystonic patterns during motor performance. In cervical dystonia, all investigations tested movement practice‐based interventions combined with other methods, such as strengthening of antagonistic muscles, postural reeducation, motor learning exercises, relaxation, stretching, massage, and functional electrical stimulation (FES).^20^, ^21^, ^22^, ^23^, ^24^

Overall, studies reported positive effects with movement practice. Significant improvements were observed in scales of disease severity, quality of life, and motor performance.^15^, ^17^, ^18^, ^23^, ^24^, ^25^ In focal hand dystonia, significant improvements were also detected in somatosensory temporal discrimination threshold and handwriting kinematics.^15^ In cervical dystonia, two randomized controlled trials reported no significant differences between specialized physical therapy and control interventions (relaxation or home‐based exercise program), even though both groups tended to show improvements over baseline.^21^, ^23^ Similarly, there were no significant differences between task‐specific training and general motor practice for focal hand dystonia; significant benefits were induced by both interventions.^15^ Investigations that compared the effects of BoNT alone with BoNT combined with rehabilitation based primarily on movement practice reported significantly lower dose and longer effects of BoNT^25^ or significant improvements in pain, ADLs, and physical and mental health.^23^, ^25^ A case study also reported decreased BoNT dose after a rehabilitation program based on intensive motor practice.^22^ The remaining studies in this category reported qualitative improvements in motor performance, pain, and quality of life in cervical^21^, ^22^, ^26^ or musician's dystonia.^16^ The GRADE level for this category was rated as low.

#### **Category 2: Training with Constraint**

Interventions in this category were based on intensive motor training with the affected body part while unaffected joints were constrained to avoid compensatory movements (*n = *7). This approach was tested exclusively in focal hand dystonia.

The term “sensorimotor retuning” has been used to describe an intervention involving immobilization by splint(s) of one or more unaffected digits.^27^ The dystonic finger was trained with repetitive exercises in coordination with one or more of the other digits. “Sensorimotor retuning” has been primarily explored in the treatment of musician's dystonia, either as the only intervention^28^, ^29^, ^30^ or in combination with other approaches.^31^, ^32^ One study in writer's cramp adapted this method to handwriting training.^33^

Training with constraint produced significant improvements in movement smoothness, speed, handwriting kinematics, and scales of motor performance and disease severity in focal hand dystonia.^27^, ^28^, ^29^, ^31^, ^32^, ^33^ Qualitative improvements in musical performance also were reported.^30^ The GRADE level for this category was rated as very low.

#### **Category 3: Sensory Reorganization**

Studies in this category have focused either on intensive sensory training or sensory deprivation to promote somatosensory reorganization and subsequent motor improvement in the dystonic body part in people with focal hand dystonia (*n = *8). This premise is based mainly on studies suggesting impaired sensory integration and decreased inhibitory mechanisms in the motor cortex of people with dystonia.^3^, ^34^ Prolonged immobilization of the affected hand and forearm was employed to induce inactivity dependent changes in sensorimotor areas in the brain.^35^, ^36^ Another approach used intensive Braille training to increase sensory discrimination of the fingers.^37^, ^38^ In sensory discriminative training^39^, ^40^ and “learning‐based sensorimotor training,”^41^, ^42^ the treatment was based on intensive sensory training of the affected hand combined with motor training, relaxation, mobilization, postural training, fitness exercises, and memory training.

Prolonged immobilization was associated with transient side effects and variable outcomes in writer's cramp and musician's dystonia.^35^, ^36^ In contrast, studies employing intensive sensory training reported significant improvements in disease severity, sensory discrimination, hand strength, and function in focal hand dystonia.^37^, ^38^, ^39^, ^40^, ^41^, ^42^ The GRADE level for this category was rated as very low.

#### **Category 4: Normalization of Muscle Activity with External Techniques**

In some sense, all interventions for focal dystonias are aimed at normalizing muscle activity to improve motor function. However, the interventions in this category focused primarily on controlling muscle activation levels using external methods or devices (*n = *9). Active approaches for normalization of muscle activity utilized primarily active participation by the patient. Active approaches included biofeedback through various methods such as visual, auditory, electromyography (EMG), or electroencephalography (EEG) readings.^43^, ^44^, ^45^, ^46^ Alternatively, passive methods were considered those in which the primary component of the intervention was the introduction of an externally applied stimulus with little or no emphasis on patient participation. Examples of passive methods included vibration, transcutaneous nerve stimulation (TENS), FES, extracorporeal shock wave therapy, and kinesiotape.^47^, ^48^, ^49^, ^50^, ^51^

Interventions based on external methods generally reported mild, but significant, improvements in handwriting (writing time and subjective scales of performance) and disease severity in individuals with writer's cramp.^45^, ^47^, ^48^ Qualitative analyses also suggested beneficial effects in writing^44^, ^46^ EMG amplitude,^44^ and discomfort and pain.^46^ Use of kinesiotape over affected muscles, a passive method for muscle activity normalization, led to significant decrease in pain and somatosensory temporal discrimination threshold in comparison to sham tape in cervical and focal hand dystonia.^50^ Extracorporeal shock wave therapy over affected muscles resulted in qualitative improvements in disease severity measured with clinical scales for musician's and focal hand dystonia.^49^ In the only study of foot dystonia, a 20% improvement was observed in the 6‐minute walk test after FES of the peroneal nerve.51 The GRADE level for this category was rated as very low.

#### **Category 5: Neuromodulation with Training**

Studies in this category tested some modality of motor training paired with neuromodulation methods (*n = *5), such as transcranial direct current stimulation (tDCS),^52^, ^53^, ^54^ repetitive transcranial magnetic stimulation (rTMS),^55^ or intermittent theta burst stimulation (iTBS).^56^ Stimulation sites tested included the premotor cortex,^55^ primary motor cortex,^52^, ^53^, ^54^ and the cerebellum.^56^

All studies in this category tested a control condition, either by including healthy participants, testing of sham or different stimulation polarities, or comparing neuromodulation with versus, without training. However, there was a large variability between studies in terms of stimulation parameters, type of motor training tested, and duration of intervention. Overall, significant improvements in musical performance, sensory discrimination, dystonia severity, and emotional well‐being were observed after motor training combined with neuromodulation in focal hand dystonia.^52^, ^53^, ^54^, ^55^ In musician's dystonia, better outcomes were reported for cathodal stimulation in comparison to other conditions,^52^, ^53^ and for real stimulation versus sham.^54^ Comparison between tDCS with and without training revealed that tDCS alone is not effective for improving motor performance in musician's dystonia.^53^ No differences were observed between rTMS followed by sensorimotor training in comparison to rTMS followed by massage and stretching in focal hand dystonia.^55^ In the only study of cervical dystonia in this category, neck motor training after real iTBS induced significant improvements in pain, quality of life, and hand dexterity in comparison to sham.^56^ The GRADE level for this category was rated as low.

#### **Category 6: Compensatory Strategies**

A few studies in writer's cramp explored the development of compensatory movement strategies with the use of a new writing technique, splinting and/or a writing device to induce changes in handwriting technique (*n = *4).^57^, ^58^, ^59^, ^60^ Although splinting or writing devices could be considered an external method (as used in studies included in Category 4), the use of devices in this category was not intended to normalize the level of muscle activity per se but rather to facilitate development of the compensatory strategy. Moreover, different than interventions focused on movement practice, the goal was to develop a different movement strategy to compensate for the dystonic impairment.

A small number of studies focused on training with compensatory strategies. Significant improvements in handwriting kinematics, writing quality, comfort, and disease severity were reported.^58^, ^59^, ^60^ However, handwriting kinematics did not match healthy individuals after an intervention based on practicing a new writing technique.^59^ The GRADE level for this category was rated as very low.

## Quality of Evidence

The overall quality of each category of rehabilitation approach ranged from very low (categories 2, 3, 4, 6)‐to‐low (categories 1 and 5) based on the GRADE system due to the study limitations identified. Study limitations included: small sample sizes, many case series or reports, lack of proper control conditions, lack of objective measures, lack of blinding or randomization, heterogeneity of participants’ characteristics in the same study, variable duration of intervention, variability in the treatment received by patients in the same study, and use of combined approaches without prior testing of isolated effects of each method. Considering the low rating for the categories of intervention, analysis of the strength of clinical recommendations, as suggested by the GRADE approach, was not conducted.

## Discussion

This systematic review is the first to classify rehabilitation studies in focal dystonias based on the theoretical basis of the interventions to help to bring together seemingly diverse approaches for improved comparison across studies. Articles published in the past 20 years were rigorously reviewed and organized into six categories based on the shared scientific underpinnings for the interventions tested. A variety of approaches and designs were employed and most were focused on adult onset idiopathic and isolated dystonia of the hand or neck; the GRADE level for each category ranged from very low‐to‐low. Despite a lack of high‐level evidence, our assessment suggests that intensive movement practice and neuromodulation combined with motor training should be further explored.

### Benefit of a Classification System

Rehabilitation studies in focal dystonia have used diverse treatment approaches making it difficult to determine if rehabilitation is effective. In defense of this diversity, the pathophysiology of the disorder is not well understood and therefore, experiments have been exploratory or Phase II trials, often driven by symptoms or based on hypothesized scientific underpinnings. However, when considered together, common themes emerge. This supports the organization of studies in dystonia rehabilitation into categories based on the theoretical basis of the interventions tested, even if diverse techniques were employed. For example, studies in Category 1 tested interventions based on intensive movement training to promote improved motor control of affected muscles, such as the use of task‐specific training in musician's dystonia.^16^, ^17^, ^18^, ^19^ In contrast, studies in Category 4 focused on improving motor performance by using external techniques such as biofeedback to facilitate the normalization of muscle activity.^43^, ^44^, ^45^, ^46^ Therefore, albeit similar, the key component of each category is distinct (i.e., movement practice vs. use of biofeedback). Utilization of the proposed classification system allows improved comparison between studies, invites further exploration for the strongest evidence, and allows future meta‐analyses. Finally, statement of a study's theoretical basis and identification of the intervention category within the classification will promote a hypothesis‐driven approach, which is essential for progress in the field. It is important to note that classification revisions will likely be needed as new rehabilitation studies are completed and new categories may be created to accommodate investigations based on a premise not yet included in the current classification.

### Limitations, Challenges and Recommendations for Rehabilitation in Focal Dystonia

Many of the studies reviewed had methodological limitations, reducing the overall strength of the evidence for each category. However, some methodological issues observed are difficult to overcome since they are typical of investigations of rare disorders such as dystonia, or represent limitations common to rehabilitation studies in general.

The most frequent limitation observed in the studies reviewed was small sample sizes. Given that each type of focal dystonia is rare and rehabilitation interventions often require multiple sessions or extensive time commitment, recruiting a statistically appropriate number of persons to participate in a lengthy study can be challenging. Several options exist to overcome this challenge. For example, rigorous single‐subject designs with multiple baselines, blinding, and randomization are a solution for overcoming small sample sizes in heterogeneous and rare disorders such as dystonia because they allow testing for both between‐subjects and within‐subject effects which are often masked by group level statistics.^61^, ^62^ Crossover designs are another suitable option for small samples, since each person receives both the experimental and the control interventions. However, carryover effects and appropriate length of the washout period must be carefully considered, especially in rehabilitation.^55^ Collective efforts such as multicenter investigations help increase patient numbers but consistency of treatment methods across different centers must be assured. Importantly, while there is disadvantage in terms of statistical power, well‐designed small sample investigations help determine which interventions have the most potential to advance to the next level of scientific inquiry, identify key outcome measures, and can help to guide clinical practice.

Other limitations observed represent common challenges faced by rehabilitation investigations in general. One common limitation was lack of proper control conditions. Primarily, there is no placebo substitute for rehabilitation (i.e., sugar pill). Some studies have used alternative interventions as a control condition, such as stretching, massage, educational sessions, or home exercises, based on their hypothesized lack of efficacy.^20^, ^21^, ^24^, ^55^ However, these methods lack similarity with the experimental treatments tested and may have produced a true benefit in the dystonia. The vital issue for designing the control condition in a rehabilitation study is to isolate the key component of the experimental intervention and keep all other aspects as similar as possible to allow statements about efficacy.

Another common challenge in rehabilitation research is determining whether the intervention should be individualized to each patient or standardized. Clinically, rehabilitation treatments are tailored to each patient's impairments and goals to increase motivation and compliance. It is known that functional improvements in chronic disorders are highly influenced by patient engagement, therefore, individualized interventions should be allowed in rehabilitation studies to some extent. Some studies reviewed employed individualized therapy.^20^, ^21^, ^40^ And, while this is laudable and practical, clinical guidelines must be included to allow for replication and transfer into clinical practice. Furthermore, considering that dystonia is a heterogeneous disorder and individuals may respond differently to the same treatment, within‐subject or sub‐group analyses this information should be reported to help identify which patient characteristics may predict favorable response.

A current issue for rehabilitation research in focal dystonia is a lack of standardized and validated outcome measures to determine functional ability or activity. There is also a need for more objective and specific assessment tools for measuring changes in disease status. For these reasons, many rehabilitation studies used custom assessment scales to quantify subjective impressions of motor performance or improvements; however, custom scales hamper comparisons across different studies and replication by other investigations. If tools have not been developed specifically for a given symptom or outcome, validated scales for assessment of subjective impressions should be used, such as the visual analogue scale, global rating of change and goal attainment scale.^63^, ^64^ Effect sizes, minimal clinically important difference and minimal detectable changes should also be established for typical measures to allow determination of the clinical significance of the findings.^63^, ^64^ Furthermore, future investigations should systematically explore the effects of combining BoNT and rehabilitation since this combination is likely to be a common approach in clinical practice. The efficacy of combining both treatments as well as changes to BoNT dose and number of injections should be measured. Considering the theoretical basis of the different rehabilitation approaches, interventions based on categories 1, 2, 4, and 6 may be the most appropriate to be combined with BoNT. Finally, future studies should include measures that allow assessment of the possible mechanisms of focal dystonia and the neurophysiological effects of each intervention in addition to efficacy measures to improve understanding of the disorder and how the treatment tested improved the condition. While the patient's primary goal is an important outcome, to only measure differences in one domain limits the potential advancement of understanding of mechanism of action or pathophysiology of the disease.

Assessments and treatment approaches of studies reviewed were evaluated according to the International Classification of Functioning, Disability, and Health (ICF).^65^ The majority of studies used impairment‐based assessments while a few evaluated patients’ activity and participation levels.^20^, ^21^, ^22^, ^23^, ^24^, ^25^, ^26^, ^39^, ^41^, ^42^, ^45^, ^50^, ^55^ Future studies need to assess participation and activity to fully detect the impact of the intervention. Furthermore, virtually every study used an impairment‐based intervention. Exploration of treatments that are aimed at improving activity and participation in addition to the impairments associated with dystonia are needed.

### Evidence Quality

The overall quality of available evidence ranged from very low‐to‐low (GRADE) due to study limitations. At this time, none of the categories of interventions explored in focal dystonia provides conclusive evidence for effectiveness of any rehabilitation approach. Therefore, specific recommendations for clinical applications are premature. However, patient report^5^, ^8^, ^9^ and promising findings by some well‐designed studies invite further investigation. Intensive movement practice and neuromodulation combined with motor training for rehabilitation of cervical and hand dystonias have the strongest level of evidence and should be further explored. It is important to recognize that it may not be possible yet for rehabilitation studies in a rare disorder to follow the medical model of large randomized placebo controlled trials. As progress is made with small‐scale studies and more conclusive evidence is gathered, larger trials may be possible through a multicenter approach. Until then, efforts should be focused on well‐designed and controlled small samples studies to help determine which approach holds the most promise.

## Author Roles

1. Research Project: A. Conception, B. Organization, C. Execution; 2. Statistical Analysis: A. Design, B. Execution, C. Review and Critique; 3. Manuscript Preparation: A. Writing the First Draft, B. Review and Critique.

C.N.P.: 1A, 1B, 1C, 3A, 3B

L.Z.: 1C, 3B

A.B.: 1C, 3B

L.B.: 1C, 3B

T.J.K.: 1A, 1C, 3B

## Disclosures

**Ethical Compliance Statement**: The authors confirm that the approval of an institutional review board was not required for this work. We have read the Journal's position on issues involved in ethical publication and affirm that this work is consistent with those guidelines.

**Funding Sources and Conflicts of Interest**: During the conception and preparation of this manuscript, C.N. Prudente was supported by the Minnesota Discovery, Research, and InnoVation Economy (MnDRIVE) initiative. L. Zetterberg has received grants from the regional agreement between Uppsala County Council and Uppsala University Hospital (ALF), Sweden. T.J. Kimberley has received a grant from the National Institute of Deafness and Other Communication Disorders (NIDCD, 1R01DC015216‐01A1).

**Financial disclosures for previous 12 months**: C.N. Prudente is currently an employee of MicroTransponder Inc. L. Zetterberg has received speaking honoraria from Allergan Pharmaceuticals International Ltd. T.J. Kimberley has received travel funds from MicroTransponder Inc.

## Supporting information


**Supplement 1**: Categories of intervention approaches

## References

[1] Defazio G . The epidemiology of primary dystonia: current evidence and perspectives. Eur J Neurol 2010;17(Suppl 1):9–14. 10.1111/j.1468-1331.2010.03053.x 20590802

[2] Steeves TD , Day L , Dykeman J , Jette N , Pringsheim T . The prevalence of primary dystonia: a systematic review and meta‐analysis. Mov Disord 2012;27:1789–1796. 23114997 10.1002/mds.25244

[3] Quartarone A , Hallett M . Emerging concepts in the physiological basis of dystonia. Mov Disord 2013;28:958–967. 23893452 10.1002/mds.25532 PMC4159671

[4] Butler AG , Duffey PO , Hawthorne MR , Barnes MP . The socioeconomic implications of dystonia. Adv Neurol 1998;78:349–358. 9750932

[5] Comella C , Bhatia K . An international survey of patients with cervical dystonia. J Neurol 2015;262:837–848. 25605434 10.1007/s00415-014-7586-2 PMC4544552

[6] De Pauw J , van der Velden K , Cox R , et al. Measuring disability in patients with cervical dystonia according to the International Classification of Functioning. Disability and Health. OTJR (Thorofare N J) 2017;37:132–140. 28621218 10.1177/1539449217697043

[7] Marsh WA , Monroe DM , Brin MF , Gallagher CJ . Systematic review and meta‐analysis of the duration of clinical effect of onabotulinumtoxinA in cervical dystonia. BMC neurology 2014;14:91. 24767576 10.1186/1471-2377-14-91 PMC4013807

[8] Valadas A , Contarino MF , Albanese A , et al. Management of dystonia in Europe: a survey of the European network for the study of the dystonia syndromes. Eur J Neurol 2016;23:772–779. 26826067 10.1111/ene.12940

[9] Silfors A , Solders G .. Living with dystonia. A questionnaire study among members of the Swedish Dystonia Patient Association. Läkartidningen 2002;99:786–789. 11894618

[10] Cogiamanian F , Barbieri S , Priori A . Novel nonpharmacologic perspectives for the treatment of task‐specific focal hand dystonia. J Hand Ther 2009;22:156–161; quiz 162. 19278828 10.1016/j.jht.2008.11.008

[11] Delnooz CC , Horstink MW , Tijssen MA , van de Warrenburg BP . Paramedical treatment in primary dystonia: a systematic review. Mov Disord 2009;24:2187–2198. 19839012 10.1002/mds.22608

[12] De Pauw J , Van der Velden K , Meirte J , et al. The effectiveness of physiotherapy for cervical dystonia: a systematic literature review. J Neurol 2014;261:1857–1865. 24413637 10.1007/s00415-013-7220-8

[13] Atkins D , Best D , Briss PA , et al. Grading quality of evidence and strength of recommendations. BMJ 2004;328:1490. 15205295 10.1136/bmj.328.7454.1490 PMC428525

[14] Guyatt GH , Oxman AD , Vist GE , et al. GRADE: an emerging consensus on rating quality of evidence and strength of recommendations. BMJ 2008;336:924–926. 18436948 10.1136/bmj.39489.470347.AD PMC2335261

[15] Zeuner KE , Peller M , Knutzen A , Hallett M , Deuschl G , Siebner HR . Motor re‐training does not need to be task specific to improve writer's cramp. Mov Disord 2008;23:2319–2327. 18816801 10.1002/mds.22222 PMC4149415

[16] Naotaka Sakai MY . Slow‐down exercise reverses sensorimotor reorganization in focal hand dystonia: a case study of a pianist. Int J Neurorehabilitation 2015;02:157.

[17] Sakai N . Slow‐down exercise for the treatment of focal hand dystonia in pianists. Med Probl Perform Art 2006;21:25–28.

[18] de Lisle R , Speedy DB , Thompson JM . Rehabilitation of a cellist whose vibrato was affected by focal dystonia. Med Probl Perform Art 2012;27:227–230. 23247881

[19] de Lisle R , Speedy DB , Thompson JM , Maurice DD . Effects of pianism retraining on three pianists with focal dystonia. Med Probl Perform Art 2006;21:105–111.

[20] Boyce MJ , Canning CG , Mahant N , Morris J , Latimer J , Fung VS . Active exercise for individuals with cervical dystonia: a pilot randomized controlled trial. Clinical rehabilitation 2013;27:226–235. 22904115 10.1177/0269215512456221

[21] Counsell C , Sinclair H , Fowlie J , et al. A randomized trial of specialized versus standard neck physiotherapy in cervical dystonia. Parkinsonism Relat Disord 2016;23:72–79. 26723272 10.1016/j.parkreldis.2015.12.010

[22] Ramdharry G . Case report: physiotherapy cuts the dose of botulinum toxin. Physiotherapy research international: the journal for researchers and clinicians in physical therapy 2006;11:117–122. 16808092 10.1002/pri.326

[23] Queiroz MA , Chien HF , Sekeff‐Sallem FA , Barbosa ER . Physical therapy program for cervical dystonia: a study of 20 cases. Funct Neurol 2012;27:187–192. 23402680 PMC3812762

[24] Smania N , Corato E , Tinazzi M , Montagnana B , Fiaschi A , Aglioti SM . The effect of two different rehabilitation treatments in cervical dystonia: preliminary results in four patients. Funct Neurol 2003;18:219–225. 15055747

[25] Tassorelli C , Mancini F , Balloni L , et al. Botulinum toxin and neuromotor rehabilitation: An integrated approach to idiopathic cervical dystonia. Mov Disord 2006;21:2240–2243. 17029278 10.1002/mds.21145

[26] Zetterberg L , Halvorsen K , Farnstrand C , Aquilonius SM , Lindmark B . Physiotherapy in cervical dystonia: six experimental single‐case studies. Physiotherapy theory and practice 2008;24:275–290. 18574753 10.1080/09593980701884816

[27] Candia V , Elbert T , Altenmuller E , Rau H , Schafer T , Taub E . Constraint‐induced movement therapy for focal hand dystonia in musicians. Lancet 1999;353:42. 10023959 10.1016/S0140-6736(05)74865-0

[28] Candia V , Schafer T , Taub E , et al. Sensory motor retuning: a behavioral treatment for focal hand dystonia of pianists and guitarists. Arch Phys Med Rehabil 2002;83:1342–1348. 12370865 10.1053/apmr.2002.35094

[29] Candia V , Wienbruch C , Elbert T , Rockstroh B , Ray W . Effective behavioral treatment of focal hand dystonia in musicians alters somatosensory cortical organization. Proc Natl Acad Sci U S A 2003;100:7942–7946. 12771383 10.1073/pnas.1231193100 PMC164692

[30] Rosset‐Llobet J , Fabregas‐Molas S . Long‐term treatment effects of sensory motor retuning in a pianist with focal dystonia. Med Probl Perform Art 2011;26:106–107. 21695359

[31] Berque P , Gray H , Harkness C , McFadyen A . A combination of constraint‐induced therapy and motor control retraining in the treatment of focal hand dystonia in musicians. Med Probl Perform Art 2010;25:149–161. 21170477

[32] Berque P , Gray H , McFadyen A . A combination of constraint‐induced therapy and motor control retraining in the treatment of focal hand dystonia in musicians: a long‐term follow‐up study. Med Probl Perform Art 2013;28:33–46. 23462903

[33] Zeuner KE , Shill HA , Sohn YH , et al. Motor training as treatment in focal hand dystonia. Mov Disord 2005;20:335–341. 15486996 10.1002/mds.20314

[34] Elbert T , Candia V , Altenmuller E , et al. Alteration of digital representations in somatosensory cortex in focal hand dystonia. NeuroReport 1998;9:3571–3575. 9858362 10.1097/00001756-199811160-00006

[35] Pesenti A , Barbieri S , Priori A . Limb immobilization for occupational dystonia: a possible alternative treatment for selected patients. Adv Neurol 2004;94:247–254. 14509680

[36] Priori A , Pesenti A , Cappellari A , Scarlato G , Barbieri S . Limb immobilization for the treatment of focal occupational dystonia. Neurology 2001;57:405–409. 11502904 10.1212/wnl.57.3.405

[37] Zeuner KE , Bara‐Jimenez W , Noguchi PS , Goldstein SR , Dambrosia JM , Hallett M . Sensory training for patients with focal hand dystonia. Ann Neurol 2002;51:593–598. 12112105 10.1002/ana.10174

[38] Zeuner KE , Hallett M . Sensory training as treatment for focal hand dystonia: a 1‐year follow‐up. Mov Disord 2003;18:1044–1047. 14502673 10.1002/mds.10490

[39] Byl NN , McKenzie A . Treatment effectiveness for patients with a history of repetitive hand use and focal hand dystonia: a planned, prospective follow‐up study. J Hand Ther 2000;13:289–301. 11129254 10.1016/s0894-1130(00)80021-6

[40] Byl NN , Nagajaran S , McKenzie AL . Effect of sensory discrimination training on structure and function in patients with focal hand dystonia: a case series. Arch Phys Med Rehabil 2003;84:1505–1514. 14586919 10.1016/s0003-9993(03)00276-4

[41] Byl NN , Archer ES , McKenzie A . Focal hand dystonia: effectiveness of a home program of fitness and learning‐based sensorimotor and memory training. J Hand Ther 2009;22:183–197; quiz 198. 19285832 10.1016/j.jht.2008.12.003

[42] McKenzie AL , Goldman S , Barrango C , Shrime M , Wong T , Byl N . Differences in physical characteristics and response to rehabilitation for patients with hand dystonia: musicians’ cramp compared to writers’ cramp. J Hand Ther 2009;22:172–181; quiz 182. 19389604 10.1016/j.jht.2008.12.006

[43] Hashimoto Y , Ota T , Mukaino M , Liu M , Ushiba J . Functional recovery from chronic writer's cramp by brain‐computer interface rehabilitation: a case report. BMC Neurosci 2014;15:103. 25179667 10.1186/1471-2202-15-103 PMC4158043

[44] O'Neill MA , Gwinn KA , Adler CH . Biofeedback for writer's cramp. Am J Occup Ther 1997;51:605–607. 9242869 10.5014/ajot.51.7.605

[45] Berger HJ , van der Werf SP , Horstink CA , Cools AR , Oyen WJ , Horstink MW . Writer's cramp: restoration of striatal D2‐binding after successful biofeedback‐based sensorimotor training. Parkinsonism Relat Disord 2007;13:170–173. 17107822 10.1016/j.parkreldis.2006.09.003

[46] Deepak KK , Behari M . Specific muscle EMG biofeedback for hand dystonia. Appl Psychophysiol Biofeedback 1999;24:267–280. 10789003 10.1023/a:1022239014808

[47] Tinazzi M , Farina S , Bhatia K , et al. TENS for the treatment of writer's cramp dystonia: a randomized, placebo‐controlled study. Neurology 2005;64:1946–1948. 15955950 10.1212/01.WNL.0000163851.70927.7E

[48] Tinazzi M , Zarattini S , Valeriani M , et al. Effects of transcutaneous electrical nerve stimulation on motor cortex excitability in writer's cramp: neurophysiological and clinical correlations. Mov Disord 2006;21:1908–1913. 16986156 10.1002/mds.21081

[49] Trompetto C , Avanzino L , Bove M , et al. External shock waves therapy in dystonia: preliminary results. Eur J Neurol 2009;16:517–521. 19187259 10.1111/j.1468-1331.2008.02525.x

[50] Pelosin E , Avanzino L , Marchese R , et al. Kinesiotaping reduces pain and modulates sensory function in patients with focal dystonia: a randomized crossover pilot study. Neurorehabil Neural Repair 2013;27:722–731. 23764884 10.1177/1545968313491010

[51] Barrett MJ , Bressman SB , Levy OA , Fahn S , O'Dell MW . Functional electrical stimulation for the treatment of lower extremity dystonia. Parkinsonism Relat Disord 2012;18:660–661. 21975264 10.1016/j.parkreldis.2011.09.017 PMC3703504

[52] Buttkus F , Baur V , Jabusch HC , Paulus W , Nitsche MA , Altenmuller E . Retraining and transcranial direct current stimulation in musician's dystonia—a case report. Mov Disord 2010;25:1758–1760. 20645404 10.1002/mds.23259

[53] Furuya S , Nitsche MA , Paulus W , Altenmuller E . Surmounting retraining limits in musicians’ dystonia by transcranial stimulation. Ann Neurol 2014;75:700–707. 24706370 10.1002/ana.24151

[54] Rosset‐Llobet J , Fabregas‐Molas S , Pascual‐Leone A . Effect of transcranial direct current stimulation on neurorehabilitation of task‐specific dystonia: a double‐blind, randomized clinical trial. Med Probl Perform Art 2015;30:178–184. 26395620 10.21091/mppa.2015.3033

[55] Kimberley TJ , Schmidt RL , Chen M , Dykstra DD , Buetefisch CM . Mixed effectiveness of rTMS and retraining in the treatment of focal hand dystonia. Front Hum Neurosci 2015;9:385. 26217209 10.3389/fnhum.2015.00385 PMC4496570

[56] Bradnam LV , McDonnell MN , Ridding MC . Cerebellar intermittent theta‐burst stimulation and motor control training in individuals with cervical dystonia. Brain Sci 2016;6:56. 10.3390/brainsci6040056 PMC5187570 27886079

[57] Waissman FQ , Pereira JS , Nascimento OJ . A new therapeutic proposal for writer's cramp: a case report. Sao Paulo Med J 2010;128:96–98. 20676577 10.1590/S1516-31802010000200010 PMC10938968

[58] Singam NV , Dwivedi A , Espay AJ . Writing orthotic device for the management of writer's cramp. Front Neurol 2013;4:2. 23372563 10.3389/fneur.2013.00002 PMC3556565

[59] Schenk T , Bauer B , Steidle B , Marquardt C . Does training improve writer's cramp? An evaluation of a behavioral treatment approach using kinematic analysis. J Hand Ther 2004;17:349–363. 15273676 10.1197/j.jht.2004.04.005

[60] Baur B , Furholzer W , Jasper I , Marquardt C , Hermsdorfer J . Effects of modified pen grip and handwriting training on writer's cramp. Arch Phys Med Rehabil 2009;90:867–875. 19406309 10.1016/j.apmr.2008.10.015

[61] Kimberley TJ , Di Fabio RP . Visualizing the effects of rTMS in a patient sample: small N vs. group level analysis. PLoS ONE 2010;5:e15155. 21151629 10.1371/journal.pone.0015155 PMC2999570

[62] Lobo MA , Moeyaert M , Baraldi Cunha A , Babik I . Single‐case design, analysis, and quality assessment for intervention research. J Neurol Phys Ther 2017;41:187–197. 28628553 10.1097/NPT.0000000000000187 PMC5492992

[63] Wewers ME , Lowe NK . A critical review of visual analogue scales in the measurement of clinical phenomena. Res Nurs Health 1990;13:227–236. 2197679 10.1002/nur.4770130405

[64] Jaeschke R , Singer J , Guyatt GH . Measurement of health status. Ascertaining the minimal clinically important difference. Control Clin Trials 1989;10:407–415. 2691207 10.1016/0197-2456(89)90005-6

[65] International classification of functioning, disability, and health: ICF. Version 1.0. Geneva: World Health Organization, [2001]; 2001.

