# Supplementary material for: Systematic Review of Rehabilitation in Focal Dystonias: Classification and Recommendations
Source: Mov Disord Clin Pract. 2018 Mar 13;5(3):237–45. doi: 10.1002/mdc3.12574 (PMC6032834; doi:10.1002/mdc3.12574)
Supplement: Supplementary file 1 — Supplement 1: Categories of intervention approaches [file MDC3-5-237-s001.docx]

**Supplement 1**: Categories of intervention approaches

| **Category 1: Movement Practice** | | | | | | |
| --- | --- | --- | --- | --- | --- | --- |
| **Study** | **Dystonia (n);**  **Controls (n)** | **Design** | **Intervention** | **Outcome Measures** | **Effects: Experimental vs. Control** | **Effects: Changes from Baseline** |
| [Boyce, Canning [20]](#_ENREF_20) | CD (20);  Healthy (0) | 2-group randomized controlled trial; blinded assessment | - Experimental: specialized PT + relaxation - Control: relaxation - 8 sessions (12w) | TWSTRS; CDQ-24; Beck Depression Inventory; cervical ROM | NS | NS |
| [Counsell, Sinclair [21]](#_ENREF_21) | CD (110);  Healthy (0) | 2-group randomized controlled trial; blinded assessment | - Experimental: specialized PT - Control: home based exercise program - 24 sessions (24w) | TWSTR; CIDP-58; EQ-5D; subjective and non-validated improvement scale | NS | No statistical analysis for within-group results, but qualitative improvements reported for TWSTRS and CIDP-58 Subgroup analysis suggested an effect of treatment center |
| [de Lisle, Speedy [19]](#_ENREF_19) | MD (3);  Healthy (0) | Case series; blinded assessment | - Motor practice with proper body biomechanics - 10 sessions (2w) | DER; subjective and non-validated performance scales | NA | Significant improvement in performance |
| [de Lisle, Speedy [18]](#_ENREF_18) | MD (1);  Healthy (0) | Case report; blinded assessment | - Vibrato training - 18 sessions | DER; subjective and non-validated performance scales | NA | Significant improvement in all measures |
| [Queiroz, Chien [23]](#_ENREF_23) | CD (40); Healthy (0) | 2-group non-randomized controlled trial; blinded assessment | - Experimental: motor learning exercises + kinesiotherapy + FES (BoNT not controlled) - Control: BoNT - 20 sessions (4w) | TWSTRS; SF-36 | Experimental: significant improvements in pain (TWSTRS), physical and mental health (SF-36) | - Experimental: significant improvements in TWSTRS and SF-36 - Control: significant improvement in motor severity subscale (TWSTRS) |
| [Ramdharry [22]](#_ENREF_22) | CD (1);  Healthy (0) | Case report | - Physical training, stretching, soft tissue mobilization, heat pack, postural advice - 14 supervised sessions + daily practice (1y) | TWSTRS | NA | Improved scores; decreased BoNT dose |
| [Sakai [17]](#_ENREF_17) | MD (20);  Healthy (0) | Case series | - “Slow-down exercise” - 1-6y | ADDS; TCS | NA | Significant improvements in TCS |
| [Naotaka Sakai [16]](#_ENREF_16) | MD (1);  Healthy (0) | Case report | - “Slow-down exercise” - 1y | Musical performance; EMG co-contraction index; STDt | NA | Improvements in all measures |
| [Smania, Corato [24]](#_ENREF_24) | CD (4);  Healthy (0) | Single subject design; crossover | - Experimental: postural reeducation - Control: biofeedback - 30 sessions (30d), 2w washout | Head realignment test; subjective and non-validated performance scale; VAS (pain) | - Experimental: significant improvement in head alignment (2/4), disability (3/4), and pain (1/2) - Control, within-subjects: significant improvement in head alignment (2/4) and disability (1/4) | Experimental + control: significant improvement in head alignment, disability and pain |
| [Tassorelli, Mancini [25]](#_ENREF_25) | CD (40);  Healthy (0) | 2-group randomized controlled trial; crossover | - Experimental: BoNT + massage + kinesiotherapy + postural education + biofeedback - Control: BoNT - Daily sessions (2w) | Tsui scale; TWSTRS; disabilities in ADLs; VAS (pain); BoNT dose and schedule | - Experimental: significant improvements in ADL and pain; significantly longer duration of benefits and lower dose of BoNT | Both groups: significant improvements in all scales after BoNT |
| [Zetterberg, Halvorsen [26]](#_ENREF_26) | CD (6);  Healthy (0) | Single subject design | - Multiple approach (strength; postural awareness; reduce movement effort and pain) - 4w | CDQ-24; VAS (pain); postural orientation index; movement energy index; TWSTRS | NA | Improvement in quality of life (5/6) and pain (3/6) |
| [Zeuner, Peller [15]](#_ENREF_15) | WC (21);  Healthy (21)* | 2-group randomized controlled trial; blinded assessment | - Experimental: task-specific training - Control: non-specific training - Immobilization prior to intervention (4w) - Daily training (8w) | WCRS; ADDS; handwriting kinematics | NS | Both groups: significant improvements in all measures |
| Quality level of evidence (GRADE): Low | | | | | | |

| *Category 2: Training with Constraint* | | | | | | |
| --- | --- | --- | --- | --- | --- | --- |
| Study | Dystonia (n);  Controls (n) | Design | Intervention | Outcome Measures | Effects: Experimental vs. Control | Effects: Changes from Baseline |
| [Berque, Gray [31]](#_ENREF_31) | MD (8);  Healthy (0) | Case series | - “Sensorimotor retuning” + “slow down exercise” - 7 sessions + daily practice (1y) | FAM; TCS; ADDS; metronome speed | NA | Significant improvement in FAM, TCS, ADDS, speed |
| [Berque, Gray [32]](#_ENREF_32) | MD (4)**;  Healthy (0) | Case series | - “Sensorimotor retuning” + “slow down exercise” - Daily practice (4y) | FAM; DES; TCS; ADDS; metronome speed | NA | Significant improvement in FAM, DES, TCS, ADDS, speed |
| [Candia, Elbert [27]](#_ENREF_27) | MD (5);  Healthy (0) | Case series | - “Sensorimotor retuning” - 8 sessions (~3h) | Finger displacement; movement smoothness; DES | NA | Significant increase in movement smoothness and DES |
| [Candia, Schafer [28]](#_ENREF_28) | MD (11);  Healthy (0) | Case series | - “Sensorimotor retuning” - 8 sessions (~3h) + daily practice (1y) | Finger displacement; movement smoothness; DES | NA | - Significant increase in movement smoothness and DES in pianists and guitarists - No changes in wind players |
| [Candia, Wienbruch [29]](#_ENREF_29) | MD (10);  Healthy (0) | Case series | - “Sensorimotor retuning” - 8 sessions (~3h) | Finger displacement; movement smoothness; DES; MEG (somatosensory finger representation) | NA | Significant increase in movement smoothness and DES; normalization of somatosensory representations which correlated with improved behavior |
| [Rosset-Llobet and Fabregas-Molas [30]](#_ENREF_30) | MD (1);  Healthy (0) | Case report | - “Sensorimotor retuning” - Daily practice (1y) | Subjective and non-validated performance scale | NA | Qualitative improvement in musical performance |
| [Zeuner, Shill [33]](#_ENREF_33) | WC (10);  Healthy (0) | Case series | - “Sensorimotor retuning” - 4-12w | FMDRS; handwriting kinematics; VAS (change in motor performance); TMS; EEG | NA | Significant increase in frequency of wrist movements and improvement in FMDRS; qualitative improvement in VAS (writing); no significant changes in cortical excitability |
| Quality level of evidence (GRADE): Very low | | | | | | |

| *Category 3: Sensory Reorganization* | | | | | | |
| --- | --- | --- | --- | --- | --- | --- |
| Study | Dystonia (n);  Controls (n) | Design | Intervention | Outcome Measures | Effects: Experimental vs. Control | Effects: Changes from Baseline |
| [Byl and McKenzie [39]](#_ENREF_39) | FHD (12);  Normative data from healthy | Case series | - Sensory discriminative training + home program (posture, relaxation, mobilization, fitness, motor imagery) - 6-18w | Sensory Integration and Praxis Test; strength; ROM; non-validated scales for posture, neural tension, balance, motor control; VAS (pain); CAFE 40; Rivermead Test of Independence | NA | Significant improvements in all variables |
| [Byl, Nagajaran [40]](#_ENREF_40) | MD (3);  Healthy (40)* | Case series; blinded assessment | - Sensorimotor training + wellness program - 19-23 sessions + daily practice | MEG (somatosensory evoked potentials);  Sensory Integration and Praxis Test; strength; ROM; Purdue Pegboard test; VAS (pain); CAFE 40 | NA | Improvement in somatosensory hand representation, target-specific performance, fine motor skills, sensory discrimination, musculoskeletal skills, functional independence |
| [Byl, Archer [41]](#_ENREF_41) | FHD (13); Normative data from healthy | Case series; blinded assessment | - “Learning based sensorimotor training”+ task practice + fitness activities+ memory training (supervised and/or home program) - 8w | Sensory Integration and Praxis Test; BCB; Digital Reaction Time Test; Tapper Test; manual muscle test;  non-validated scale for posture; CAFE 40 | NA | Significant improvements in task specific performance, sensory discrimination,  fine motor speed, functional independence, strength |
| [McKenzie, Goldman [42]](#_ENREF_42) | MD (14) vs.  WC (13);  Healthy (0) | 2-group case series | - “Learning based sensorimotor training” + education on healthy habits + home program - 8w | Sensory Integration and Praxis Test; strength; ROM; non-standard scales for posture, neural tension, Digital Reaction Time; Motor Control Test; Motor Accuracy Test; CAFE 40 | - MD: significantly better posture, ROM, graphestesia - WC: significantly better strength and kinesthesia | Both groups: significant improvements in posture, sensory discrimination, motor control and writing speed |
| [Pesenti, Barbieri [35]](#_ENREF_35) | MD (15) +  WC (4); Healthy (0) | Case series | - Hand and forearm immobilization with splint - Daily (4-5w) | Subjective and non-validated performance scale; hand grip test | NA | Transient side effects; variable outcomes at follow-up |
| [Priori, Pesenti [36]](#_ENREF_36) | MD (7) +  WC (1); Healthy (0) | Case series | - Hand and forearm immobilization with splint - Daily (4-5w) | ADDS; TCS; subjective and non-validated performance scale | NA | Transient side effects; variable outcomes at follow-up |
| [Zeuner, Bara-Jimenez [37]](#_ENREF_37) | WC (10);  Healthy (10) | 2-group, non-randomized controlled trial | - Braille training - ~1h daily (8w) | FMDRS; GOT; time to write a standard paragraph; verbal scale and VAS (subjective improvement) | Not reported | - WC: significant improvements in FMDRS - Both groups: no difference in GOT at baseline; significant improvements in GOT |
| [Zeuner and Hallett [38]](#_ENREF_38) | WC (3)***; Healthy (0) | Case series | - Braille training - Daily (1y) | FMDRS; GOT; time to write a standard paragraph; verbal scale and VAS (subjective improvement) | NA | Improvements in GOT, time to write a standard paragraph, and verbal scale |
| Quality level of evidence (GRADE): Very low | | | | | | |

| *Category 4: Normalization of Muscle Activity with External Techniques* | | | | | | |
| --- | --- | --- | --- | --- | --- | --- |
| Study | Dystonia (n);  Controls (n) | Design | Intervention | Outcome Measures | Effects: Experimental vs. Control | Effects: Changes from Baseline |
| [Barrett, Bressman [51]](#_ENREF_51) | Foot (1);  Healthy (0) | Case report | - FES of peroneal nerve - 18 months (daily use) | 6-minute walk test; single leg stance time; TUG | NA | 20% improvement in 6-minute walk; increased time on single leg stance; increased time on TUG (worse) |
| [Berger, van der Werf [45]](#_ENREF_45) | WC (5);  Healthy (16)* | Case series | - EMG biofeedback + sensorimotor training - 5-10 sessions (2w or monthly) | D2-receptor binding (SPECT), number of graphemes/min below 500μV | NA | Significant improvement in handwriting (increased graphemes) and increase in D2-binding |
| [Deepak and Behari [46]](#_ENREF_46) | WC (10);  Healthy (0) | Case series | - EMG biofeedback (auditory) + daily writing practice - Minimum of 4 sessions over 8w | VAS (handwriting, discomfort and pain) | NA | 38-93% improvement in VAS in 9/10 patients; no effects in one patient |
| [Hashimoto, Ota [43]](#_ENREF_43) | WC (1);  Healthy (0) | Case report | - EEG biofeedback + motor training - 10 sessions (5 mo) | EEG changes, subjective analysis of handwriting | NA | Significant decrease of beta frequency, no clear clinical change |
| [O'Neill, Gwinn [44]](#_ENREF_44) | WC (1);  Healthy (0) | Case report | - EMG biofeedback (visual and auditory) + daily handwriting practice at home - 2 sessions | Self-report of symptoms, EMG amplitude | NA | Improved writing (self-report); decrease in EMG amplitude (absolute values); symptoms relapsed 4 weeks after treatment |
| [Pelosin, Avanzino [50]](#_ENREF_50) | CD (14);  FHD (11);  Healthy (0) | 2-group randomized controlled trial; crossover; single blinded | - Experimental: kinesiotape - Control: sham tape - 2w, 4w washout | VAS (pain); TWSTRS; WCRS; STDt | Experimental: significantly decreased pain and STDt (affected area) | - CD, experimental: significant decrease in pain and STDt for the neck - FHD, experimental: significant decrease in pain and STDt for the forearm - Control: no changes |
| [Tinazzi, Farina [47]](#_ENREF_47) | WC (10);  Healthy (0) | Randomized controlled trial; cross-over; single blinded | - Experimental: TENS over forearm flexor muscles - Control: placebo ultrasound - 10 sessions (2w), 12w washout | Non-standard dystonia movement scale; time to write a standard paragraph; VAS and verbal (subjective improvement) | Experimental: significant improvement in writing time, VAS and verbal scale scores | - Experimental: significant improvement in writing time, VAS and verbal scale scores - Control: no changes |
| [Tinazzi, Zarattini [48]](#_ENREF_48) | WC (10);  Healthy (14) | 2-group, non-randomized, controlled trial | - TENS over 2 forearm muscles - Conditions: 1 and 15 sessions - 3w | MEP amplitude; time to write a standard paragraph | - 1 session: significant changes to MEP amplitude in WC in comparison to controls - 15 sessions: no differences in MEP amplitude between groups | - WC: no effects after 1 session; significant changes to MEP amplitude and reduction in writing time after 15 sessions; no significant correlation between changes in MEP amplitude and writing time - Healthy: significant changes to MEP amplitude after 1 and 15 sessions |
| [Trompetto, Avanzino [49]](#_ENREF_49) | FHD (3)**** + WC (3) | Case series; crossover design; single blinded | - Experimental: Extracorporeal Shock Wave Therapy over affected muscles - Control: placebo shock - 4 sessions (4w) | UDRS; ADDS; electrophysiological measures (somatosensory evoked potentials, compound motor action potential, F wave) | Experimental: UDRS and ADDS scores decreased in some patients; no electrophysiological changes | - Experimental: better outcomes in secondary dystonia - Control: no changes |
| Quality level of evidence (GRADE): Very low | | | | | | |

| *Category 5: Neuromodulation with Training* | | | | | | |
| --- | --- | --- | --- | --- | --- | --- |
| Study | Dystonia (n);  Controls (n) | Design | Intervention | Outcome Measures | Effects: Experimental vs. Control | Effects: Changes from Baseline |
| [Buttkus, Baur [52]](#_ENREF_52) | MD (1);  Healthy (0) | Single subject design; crossover; double blinded | - “Slow down exercise” + tDCS over M1 - Conditions: cathodal, anodal or sham - 5 sessions/ condition, 6w washout | MIDI-based scale | Cathodal tDCS: significantly better performance than other conditions | All conditions: significant improvements in MIDI-based scale; most prominent effects immediately after retraining |
| [Furuya, Nitsche [53]](#_ENREF_53) | MD (10);  Healthy musicians (10) | 2-group within-subject randomized, crossover controlled trial; double blinded | - Bimanual mirrored finger movements + tDCS over M1 - Conditions: cathodal, anodal, sham, unihemispheric, or cathodal without training - 5 sessions, 15d washout | Rhythmic variability of sequential finger movements | Cathodal tDCS + training: significant improvements | - Cathodal tDCS+ training: significant improvements in affected hand - Other conditions: no changes - Healthy: no changes |
| [Rosset-Llobet, Fabregas-Molas [54]](#_ENREF_54) | MD (30);  Healthy (0) | Randomized controlled trial; double blinded | - Experimental: “sensorimotor retuning”+ real tDCS (M1) - Control: “sensorimotor retuning”+ sham tDCS (M1) - 2w | Non-standard dystonia severity rating; subjective and non-validated performance scale | Experimental: significant improvement in dystonia severity and musical performance | Experimental and control: significant improvements in dystonia severity |
| [Kimberley, Schmidt [55]](#_ENREF_55) | FHD (9);  Healthy (0) | Randomized single subject design; crossover | - Experimental: real rTMS (premotor cortex) + “learning-based sensorimotor training” - Control: real rTMS (premotor cortex) + stretching + massage - 5 sessions, 30d washout | GROC; ADDS; SF-36; handwriting (kinematics & video); STDt; BCB test; cortical excitability (TMS) | NS; carry-over effects after both interventions | Experimental + control: significant improvements in emotional well-being, ADDS, STDt, BCB |
| [Bradnam, McDonnell [56]](#_ENREF_56) | CD(16);  Healthy (0) | Randomized controlled trial; double blinded | - Experimental: real iTBS (cerebellum) + neck motor training + implicit learning task - Control: sham iTBS (cerebellum) + neck motor training + implicit learning task - 10 sessions | TWSTRS, CDQ-24, Grooved Pegboard Test (hand dexterity), upper trapezius MEP amplitude and cortical silent period | Experimental: significant improvement in TWSTRS (pain subscale), CDQ-24, and Grooved Pegboard Test | Experimental: significant improvement in TWSTRS (total score and pain subscale), CDQ-24, and Grooved Pegboard Test |
| Quality level of evidence (GRADE): Low | | | | | | |

| *Category 6: Compensatory Strategies* | | | | | | |
| --- | --- | --- | --- | --- | --- | --- |
| Study | Dystonia (n);  Controls (n) | Design | Intervention | Outcome Measures | Effects: Experimental vs. Control | Effects: Changes from Baseline |
| [Baur, Furholzer [60]](#_ENREF_60) | WC (26); Healthy (14)* | Non-randomized controlled trial | - Modified pen grip + handwriting exercises - 7 sessions | Handwriting (grip force, pressure, frequency, fluency); FMDRS; VAS (impairment and pain) | NA | Significant decrease in pressure and grip force; significant improvements in FMDRS and VAS |
| [Schenk, Bauer [59]](#_ENREF_59) | WC (50);  Healthy (21)* | Case series | - New handwriting technique - 2-20 sessions | Handwriting kinematics (speed, number of letters, peak velocity per stroke) | NA | Significant improvement in handwriting kinematics (but it did not match controls at posttest) |
| [Singam, Dwivedi [58]](#_ENREF_58) | WC (15);  Healthy (0) | Case series | - Training with orthotic device that modifies handwriting posture - Daily practice (2w) | WCRS; VAS (writing quality and comfort) | NA | Significant improvement in WCRS; improved writing quality and comfort |
| [Waissman, Pereira [57]](#_ENREF_57) | WC (2);  Healthy (0) | Case series | - Body awareness + immobilization of affected muscles with splints + writing retraining - 16 sessions + daily home practice (8w) | FMDRS; Jedynak Evaluation; VAS (pain) | NA | Qualitative improvements in all measures |
| Quality level of evidence (GRADE): Very low | | | | | | |

*Group included for comparisons only; these participants did not receive any intervention.

**All subjects also participated in the study from [Berque, Gray [31]](#_ENREF_31).

***All subjects also participated in the study from [Zeuner, Bara-Jimenez [37]](#_ENREF_37).

****Participants with FHD had dystonia secondary to a lesion in the basal ganglia. The remaining participants had idiopathic WC.

Abbreviations: ADDS: Arm Dystonia Disability Scale; ADLs: activities of daily living; BCB: Byl–Cheney–Boczai sensory discrimination test; BoNT; botulinum neurotoxin; CAFE 40: California Functional Evaluation 40; CD: cervical dystonia; CDQ-24: Craniocervical Dystonia Questionnaire; CIDP-58: Cervical Dystonia Impact Profile; CTL: controls; d: days; DER: Dystonia Evaluation Rating; DES: Dystonia Evaluation Scale; EEG: electroencephalography; EMG: electromyography; EQ-5D: Euro quality of life five dimensions questionnaire; FAM: Frequency of Abnormal Movements Scale; FES: functional electrical stimulation; FHD: focal hand dystonia; FMDRS: Fahn-Marsden Dystonia Rating Scale; GOT: Grating Orientation Discrimination Test; GROC: global rating of change; h: hour; iTBS: intermittent theta burst stimulation; M1: primary motor cortex; MD: musician’s dystonia; MEG: magnetoencephalography; MEP: motor evoked potential; MIDI: musical instrument digital interface; mo: months; NA: not applicable; NS: not significant; PT: physical therapy; ROM: range of motion; rTMS: repetitive transcranial magnetic stimulation; SF-36: 36-Item Short Form Survey; SPECT: single-photon emission computed tomography; STDt: somatosensory temporal discrimination threshold; TCS: Tubiana and Champagne Dystonia Scale; tDCS: transcranial direct current stimulation; TENS: Transcutaneous Electrical Nerve Stimulation; TMS: transcranial magnetic stimulation; TUG: Timed Up and Go test; TWSTRS: Toronto Western Spasmodic Torticollis Rating Scale; UDRS: Unified Dystonia Rating Scale; VAS: visual analog scale; w: weeks; WC: writer’s cramp; WCRS: Writer's Cramp Rating Scale; y: year.
